# Exploring the role of ChatGPT in patient care (diagnosis and treatment) and medical research: A systematic review

**DOI:** 10.34172/hpp.2023.22

**Published:** 2023-09-11

**Authors:** Ravindra Kumar Garg, Vijeth L Urs, Akshay Anand Agarwal, Sarvesh Kumar Chaudhary, Vimal Paliwal, Sujita Kumar Kar

**Affiliations:** ^1^Department of Neurology, King George’s Medical University, Lucknow, India; ^2^Department of Surgery, King George’s Medical University, Lucknow, India; ^3^Department of Neurology, Sanjay Gandhi Institute of Medical Sciences, Lucknow, India; ^4^Department of Psychiatry, King George’s Medical University, Lucknow, India

**Keywords:** Artificial intelligence, Machine learning, Authorship, Publishing, Scholarly

## Abstract

**Background:**

ChatGPT is an artificial intelligence based tool developed by OpenAI (California, USA). This systematic review examines the potential of ChatGPT in patient care and its role in medical research.

**Methods:**

The systematic review was done according to the PRISMA guidelines. Embase, Scopus, PubMed and Google Scholar data bases were searched. We also searched preprint data bases. Our search was aimed to identify all kinds of publications, without any restrictions, on ChatGPT and its application in medical research, medical publishing and patient care. We used search term "ChatGPT". We reviewed all kinds of publications including original articles, reviews, editorial/ commentaries, and even letter to the editor. Each selected records were analysed using ChatGPT and responses generated were compiled in a table. The word table was transformed in to a PDF and was further analysed using ChatPDF.

**Results:**

We reviewed full texts of 118 articles. ChatGPT can assist with patient enquiries, note writing, decision-making, trial enrolment, data management, decision support, research support, and patient education. But the solutions it offers are usually insufficient and contradictory, raising questions about their originality, privacy, correctness, bias, and legality. Due to its lack of human-like qualities, ChatGPT’s legitimacy as an author is questioned when used for academic writing. ChatGPT generated contents have concerns with bias and possible plagiarism.

**Conclusion:**

Although it can help with patient treatment and research, there are issues with accuracy, authorship, and bias. ChatGPT can serve as a "clinical assistant" and be a help in research and scholarly writing.

## Introduction

 ChatGPT (Chat Generative Pre-trained Transformer) is an artificial intelligence (AI) based on a natural language processing tool developed by OpenAI (California, USA). ChatGPT is chat boat based technology. A chatbot is in fact a type of software creates text akin to human-like conversation. ChatGPT has the capacity to respond to follow-up questions, recognise errors, debunk unfounded theories, and turn down inappropriate requests.large language models (LLMs), which are frequently abbreviated as LLMs, are extremely complex deep-learning programmes that are capable of comprehending and producing text in a manner that is strikingly comparable to that of humans.LLMs can recognise, summarise, translate, predict, and create text as well as other sorts of information by using the large knowledge base they have amassed from massive datasets.^1-4^

 The possible uses of ChatGPT in medicine is currently under intense investigation. ChatGPT is considered to have enormous capability in helping experts with clinical and laboratory diagnosis to planning and execution of medical research.^5,6^ Another significant use of ChatGPT in medical researchers is the creation of virtual assistants to physicians helping them in writing manuscripts in more efficient way.^7^ Usage of ChatGPT in medical writing is considered to have associated with several ethical and legal issues. Possible copyright violations, medical-legal issues, and the demand for openness in AI-generated content are a few of these.^8-12^

 The accuracy of ChatGPT in producing trustworthy health information, the ethical and legal ramifications, the interpretability of AI decisions, the potential for bias, the integration with healthcare systems, professional AI literacy, patient perspectives, and data privacy issues are some of the key knowledge gaps about ChatGPT’s role in medical research and clinical practise.^13,14^ In this systematic review we aimed to review published article and explore the potential of ChatGPT in facilitating patient care, medical research and medical writing. We will also focus on ethical issues associated with usage of ChatGPT.

## Materials and Methods

 We performed a systematic review of published articles on ChatGPT. The protocol of the systematic review was registered with PROSPERO (CRD42023415845).^15^ Our systematic review was conducted following the Preferred Reporting Items for Systematic Reviews and Meta-Analyses (PRISMA) guidelines.

###  Search strategy

 We searched four databases, PubMed, Scopus, Embase, and Google Scholar. Our search was aimed at identifying all kinds of articles on ChatGPT and its application in medical research, scholarly and clinical practice, published till 24 May 2023. Articles related to medical education was not considered. The search item that we used was “ChatGPT”. We reviewed all kinds of publications including original articles, reviews, editorial/ commentaries and even letter to the editor describing ChatGPT. We did not put any kind of restriction or limitations in our search strategy.

###  Data extraction

 The selection of the papers that were published was done in two steps. Two reviewers (RKG and VP) reviewed the titles and abstracts in the initial phase. Two reviewers (VLU and SKC) then examined the entire texts of the chosen papers to determine their eligibility. A third author (SKC) settled any differences that arose between the two authors. Two reviewers (RKG and VP) assessed the information available in the included publication for the suitability of the article to be included in the review. Any disagreement between them was resolved by mutual agreement. If a dispute persisted, it was resolved via consultation with a third reviewer (SKC).

 EndNote 20 web tool (Clarivate Analytics) was used to handle duplicate records. This process was carried out by two reviewers independently (RKG and VP). Any issue that arose was resolved with a discussion with another reviewer. The number of retrieved and assessed records at each stage was provided in the form of a PRISMA flow chart. EndNote 20 (Clarivate Analytics) was used to make a PRISMA flow chart.

 The reviewers involved in this review are faculty in a leading teaching institution of India. They all have sufficient training and knowledge systematic review procedures.

###  Data analysis

 Data analysis was done jointly by two reviewers (RKG and VP). ChatGPT was extensively used for analysing the selected records and writing this manuscript. A table was made with six columns (First author/sole author, country of origin, status of peer review (peer-reviewed or preprint), title of the paper and short point wise summary of full text. Short point wise summary of full text of each and every article was created with the help of ChatGPT. The voluminous word file was then converted to a pdf file and was processed with the sister software “ ChatPDF” (OpenAI, California, USA available at https://www.chatpdf.com/). Following questions were asked from ChatPDF.

1. What are potential role of ChatGPT in medical writing and research?
2. What could be the role of ChatGPT in clinical practice?
3. What are ethical issues associated with paper writing?
4. Can ChatGPT be an author?
5. Can ChatGPT write text in good English and free of plagiarism?
6. Role of ChatGPT so far in neurological disorders related clinical practice and research.
7. Effectiveness and efficiency of ChatGPT in medical research and clinical settings
8. Potential benefits and limitations of ChatGPT in medical research and clinical applications
9. The ethical implications of using ChatGPT in medical research and clinical practice
10. Identify the gaps in the current research on ChatGPT and suggest areas for further investigation.
11. Provide insights into the potential future applications of ChatGPT in medical research and clinical practice
12. Recommendations for researchers, clinicians, and policymakers on the use of ChatGPT in medical research and clinical practice

All the responses were compiled in a word file.

###  Quality assessment

 Quality assessment was not done.

## Results

**Figure 1:**
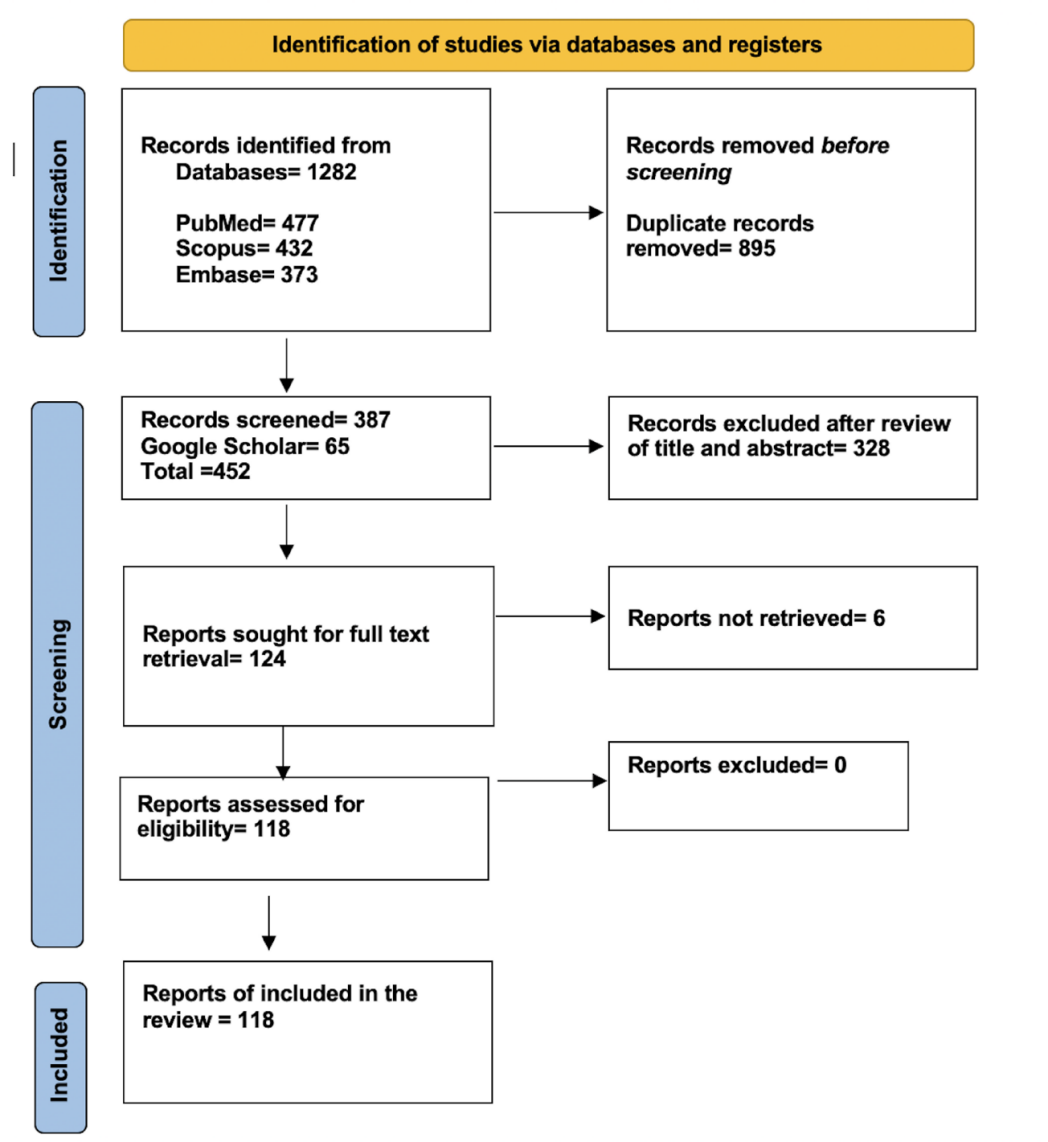
The study's PRISMA flow diagram shows how articles are selected for this systematic review

 Our data collection followed PRISMA guidelines (Table S1, Supplementary file 1) The PRISMA flowchart for our systematic review is shown in Figure 1. We reviewed 118 publications. ChatGPT related publications are available from across the globe. There were 33 original articles and rest were commentary/editorial, review articles, research letters or letter to the editors. Out of 118 articles, 18 articles were available as preprint only. Summaries of 118 articles and answers to 12 questions have been provided in form of tables.^16-133^ (Supplementary file 2, Table S2 and S3).

 The multiple dimensions of ChatGPT’s role in medical research include assisting with data gathering, analysis, and interpretation, assisting with scientific writing and publication editing, assisting with decision-making and treatment planning, and enhancing medical teaching and learning. In order to increase the effectiveness of data collection and processing, ChatGPT can be used to speed up procedures like patient questionnaires, interviews, and epidemiological research. Additionally, it can assist researchers in locating essential information, developing hypotheses, and analysing data—all of which will quicken the research process. In order to generate ideas, create articles, and support authors in creating clear and understandable content, ChatGPT can be utilised in scientific writing. Text summary, language editing, and proofreading, even for abstracts, can all be aided by it. To ensure quality, it’s crucial to thoroughly assess and edit the content produced by ChatGPT.

 Additionally, ChatGPT can be employed as a valuable tool in clinical practice. It can assist clinicians in patient inquiries, writing medical notes and discharge summaries, and making informed decisions about treatment plans. It has the potential to serve as a personalized learning tool, encouraging critical thinking and problem-based learning among medical professionals.

 While ChatGPT offers numerous benefits, there are also limitations and ethical considerations to be addressed. These include potential biases in training data, issues of accuracy and reliability, privacy concerns, questions about authorship in academic papers, and ethical implications of its use. It is crucial to establish regulations and control mechanisms to ensure the ethical utilization of ChatGPT and similar AI tools in medical research and clinical practice.

## Discussion

 We looked into two main uses of ChatGPT: in healthcare settings and for medical writing and research. We studied 118 articles - most were opinion pieces, commentaries, and reviews.^16-133^ Another group, Ruksakulpiwat et al, also did a similar study. They analyzed six articles out of 114 that met their criteria. These articles covered a variety of ways to use ChatGPT, such as finding new drugs, writing literature reviews, improving medical reports, providing medical info, bettering research methods, analyzing data, and personalizing medicine.^134^

 Levin et al, on the other hand, conducted an analysis of the first batch of publications about ChatGPT. They found 42 articles published in 26 journals in the 69 days after ChatGPT was launched. Only one was a research article. The rest were mostly editorials and news pieces. Five publications focused on studies on ChatGPT. There were no articles on its use in Obstetrics and Gynecology. In terms of where these articles were published, Nature was the top journal. Radiology and Lancet Digital Health came next. The articles mostly discussed the quality of ChatGPT’s scientific writing, its features, and its performance. Some also talked about who should get credit for the work and ethical concerns. Interestingly, when comparing the articles that described a study to the others, the average impact factor (a measure of the influence of a journal) was significantly lower for the study articles.^135^

 In our review, we identified several potential advantages of using ChatGPT in the medical field. It appears to enhance productivity and expedite research workflows by aiding in data organization, assisting in the selection of trial candidates, and supporting overall research activities. Furthermore, ChatGPT’s capacity to review manuscripts and contribute to editing may potentiate the efficiency of academic publishing.^136^ Beyond the scope of research, it could also prove beneficial for patient education, fostering scientific exploration, and shaping clinical decision-making.^137^ However, we also need to consider certain limitations and ethical concerns associated with the use of ChatGPT. The model, as sophisticated as it is, lacks the capability to offer comprehensive diagnoses and cannot replace the human qualities inherent to medical practice.^138^ Ethical issues also arise, specifically in relation to potential biases in the machine learning model and potential breaches of privacy.^139,140^ Moreover, while ChatGPT can process and generate information, it might not exhibit the level of originality, creativity, and critical thinking that are often required in the medical field. However, the use of ChatGPT in producing scholarly articles is raising questions in the academic publishing. While these tools can greatly enhance the clarity and fluency of written material, it is crucial that human oversight is maintained throughout the process. This is because AI can potentially produce content that is authoritative-sounding, yet it might be inaccurate, incomplete, or biased. Incorrect GPT-4 responses, known as “hallucinations,” can be harmful, particularly in the field of medicine.^22,141^ Therefore, it is essential to check or validate GPT-4’s output. ChatGPT can generate references to made-up research publications.^142^ Therefore, authors must thoroughly check and modify the output of these tools. Furthermore, it is not appropriate to recognize AI or AI-assisted tools as authors or co-authors in the by-line of publications. Instead, their use should be transparently acknowledged within the manuscript.^143,144^ For example, according to Elsevier’s policy on AI for authors, the responsibility and accountability for the work ultimately still lie with the human authors, despite any technological assistance they may have received.^145^ Authors who wish to use ChatGPT for publishing medical content should comply with the specific regulations set by the journal pertaining to AI-generated contents.

## Limitations

 There are certain limitations to our systematic review on ChatGPT. The search term used for the systematic review was “ChatGPT”. This could limit the search as not all articles might use this exact term when discussing or evaluating the tool. Variations such as “OpenAI’s language model”, “GPT-4”, or other related terms could have been included to increase search specificity. Our study does not consider articles related to medical education. This could limit the scope of the review, as ChatGPT might have potential applications and limitations within the field of medical education that are not captured. Our review only analyzes ChatGPT and doesn’t compare it to other AI models or tools that could be used in a similar capacity. This might limit the understanding of where ChatGPT stands relative to other comparable AI technologies. As of the date the systematic review was conducted, there may not have been many long-term original studies available regarding the use of ChatGPT in medical research and patient care. This could limit the review’s ability to provide a complete picture of the tool’s effectiveness and potential issues over time. Our review includes all kinds of publications such as editorials, letters to the editor and preprints which may not be peer-reviewed or have rigorous methodologies. This might affect the quality of evidence used for this review.

 In conclusion, ChatGPT has a great potential. Its full potentials are still evolving. ChatGPT as a source of information cannot be trusted, many ethical issues are associated with it. Certainly, ChatGPT cannot be credited with authorship. However, ChatGPT is certainly a good clinical assistant. ChatGPT is nowhere near to replace human brain. Before deploying in a clinical setting, it is essential to ensure that the model can provide accurate and reliable information. The ChatGPT model should be continually updated and improved based on feedback from its users to rectify its limitations. Clear guidelines need to be developed for healthcare professionals and patients on when and how to use ChatGPT as a tool. Policies need to be created to protect patient data and privacy. Ethical guidelines should be developed to address the moral dilemmas that arise from using ChatGPT in healthcare.

## Acknowledgments

 The concept, data collection analysis, writing, and reporting of this article were solely done by authors. ChatGPT was extensively utilized as mentioned in the methods section.

## Competing Interests

 None.

## Ethical Approval

 None.

## Funding

 None.

## Supplementary Files


Supplementary file 1(PRISMA check list) contains Table S1.
